# Prevalence of dysfunctional but viable myocardium in patients with ischemic cardiomyopathy - results from clinical scans performed in 2010-2014 at four U.S. hospitals

**DOI:** 10.1186/1532-429X-18-S1-O1

**Published:** 2016-01-27

**Authors:** Aditya Mandawat, Anant Mandawat, Anna Lisa Crowley, John Heitner, Alexander Ivanov, Dipan J Shah, Faisal Nabi, Afshin Farzaneh-Far, Han W Kim, Wolfgang G Rehwald, Raymond Kim, Robert Judd, Igor Klem

**Affiliations:** 1 grid.189509.c0000000100241216Cardiology, Duke University Hospital, Durham, NC USA; 2 grid.415436.10000000404437314New York Methodist Hospital, Brooklyn, NY USA; 3 grid.63368.380000000404450041Houston Methodist DeBakey Heart & Vascular Center, Houston, TX USA; 4 grid.185648.60000000121750319University of Illinois at Chicago, Chicago, IL USA; 5 Siemens Healthcare, Chapel Hill, NC USA

## Background

Assessment of viability in patients with ischemic cardiomyopathy (ICM) prior to possible revascularization has fallen out of favor after the STITCH trial, which failed to demonstrate a benefit of echocardiography or nuclear viability testing. CMR assessment of viability by directly visualizing the transmural extent of both viable and non-viable myocardium offers unique advantages which have not been explored in large trials. Several small studies have shown significant contractile improvement after revascularization of dysfunctional segments with residual viability by CMR. The objective of this study was to determine the prevalence of residual viability over the entire range of severity of dysfunction in patients with ICM.

## Methods

Data analysis was performed on a cloud-based system that is currently receiving de-identified searchable data from electronically-signed clinical reports with full DICOM datasets for 23,275 consecutive CMR exams performed at four U.S. hospitals from Jan 1, 2010 through Dec 31, 2014. At the time of analysis 8,242 datasets were available, and analysis of all 23,275 is expected by the end of 2015. All data were derived from CMR reports that had been electronically signed by board-certified physicians with Level 3 CMR training. ICD-9 codes (410, 411, 412, 413, 414) were used to identify patients with a history of coronary artery disease. Regional contractility (normal, mild/moderate hypokinesia, severe hypokinesia, akinesia, dyskinesia) and transmural extent of hyperenhancement (0%, 1-25%, 26-50%, 51-75%, 76-100%) were quantified using a standard seventeen-segment model. Significant viability was defined as ≤50% transmural extent of hyperenhancement.

## Results

Of the 1,763 patients with coronary artery disease undergoing CMR, 1,195 patients had chronic ischemic heart disease. Among these, 218 (18%) patients had a left ventricular ejection fraction ≤40% (median 30) and were included in the subsequent analyses. The median age of these patients was 66 years (IQR 16 years), 57% were male, 28% had a history of diabetes mellitus, 56% had a history of hypertension, and 49% had a history of hyperlipidemia, 15% were active smokers. In total 3,706 segments were analyzed, of which 2,898 (78%) had abnormal contractility. Among all dysfunctional segments, 2,516 segments (87%) showed significant viability (Table 1). Even among the 1,709 segments with severe dysfunction (severe hypokinesia, akinesia, dyskinesia) 1,338 segments (78%) showed significant viability.

**Table 1:** Severity of segmental dysfunction according to presence or absence of viable myocardium

	Severity of Segmental Dysfunction			
Hyperenhancement	Mild/Moderate Hypokinesia (N = 1189)	Severe Hypokinesia (N = 1058)	Akinesia (N = 535)	Dyskinesia (N = 116)
0-50%	1178 (99%)	1000 (95%)	302 (56%)	36 (31%)
51-100%	11 (1%)	58 (5%)	233 (44%)	80 (69%)

## Conclusions

A significant proportion of severely dysfunctional segments in patients with ICM demonstrated residual viability as assessed by CMR, which was shown previously to correlate with a high degree of functional improvement after revascularization. The utility of viability assessment by CMR in guiding revascularization in patients with ICM should be revisited.

